# A New Biological Product Shows Promising Control of the Northern Root-Knot Nematode, *Meloidogyne hapla*, in Greenhouse Tomatoes

**DOI:** 10.2478/jofnem-2023-0023

**Published:** 2023-05-26

**Authors:** Elisabeth Darling, Abigail Palmisano, Henry Chung, Marisol Quintanilla-Tornel

**Affiliations:** Department of Entomology, Michigan State University, 288 Farm Ln, East Lansing, MI, 48843, USA

**Keywords:** biological control, management, *Meloidogyne hapla*, nematicides, tomatoes

## Abstract

Tomato plants are susceptible to significant yield losses when infested by the northern root-knot nematode, *Meloidogyne hapla.* While there are many options for conventional chemical management, few of these options offer effective control for organic growers or those who seek to adopt more environmentally considerate strategies. In this study, we showed that a new, biologically based product (referred to as “MN21.2”) has potential for controlling populations of the northern root-knot nematode, *Meloidogyne hapla*, as a pest of susceptible tomato (cv. Rutgers) in a greenhouse trial. This is significant because if this product’s efficacy is supported under field conditions, it may provide organic tomato growers with a valuable tool for fighting the plant-parasitic nematode pest, *M. hapla.*

There are many challenges that the tomato industry faces, from the fragility of the crop itself in a changing climate, to its susceptibility to damage from insect and soilborne pests, and to its production techniques that add stress to natural resources and ecosystem services (Lange and Bronson, 1981; Boulard et al., 2011; Pathak and Stoddard, 2018). Tomatoes are one of the most widely grown and valuable crops produced in the United States and take the lead in consumption per capita of processed vegetables (Gould, 2014). Tomatoes contain high levels of antioxidants important for human consumption, including vitamin C, polyphenols, and carotenoids, levels of which vary depending on variety, ripeness, and cultivation (Hallmann, 2012). Prior to the 1780s, tomatoes were produced and consumed mainly in European countries and did not peak in popularity in the U.S. until 1830-1840 (Gould, 2014). Of the 170 million tons of tomatoes produced worldwide, the United States contributes significantly to worldwide production, as the second top producer of fresh and processing tomatoes, after China (Guan et al., 2018).

The northern root-knot nematode (NRKN), *Meloidogyne hapla*, is a significant pest that affects growth and yield in tomato production. *M. hapla* are infamous for their ability to cause major yield losses in many northern United States vegetable crops like lettuce, carrots, and tomatoes (Yeates, 1987; Chen et al., 1999; Gugino et al., 2006). *M. hapla* are sedentary endoparasitic nematodes that enter plant root material as juveniles and can cause significant root galling during their reproduction process as mature females develop. Severe infestations by *M. hapla* typically result in galled root systems, stunted plant height, increased root weight, and decreased top weight, resulting in 40% loss of fruit production (Olthof and Potter, 1977; Gugino et al, 2006). Due to the tomato’s international importance and its vulnerability to this nematode, it is vital to study how infestations can negatively impact yield of this crop and potential routes of management. This will lead to increased information on how to effectively manage infestations and limit economic losses.

Historically, the leading management process to combat nematode infestations was chemical control, such as the fumigant methyl bromide (Cetintas and Yarba, 2010; Desaeger et al., 2020). Due to the increasing regulations and push for less toxic chemicals, scientists and farmers alike are seeking economically feasible and environmentally responsible alternatives for root-knot nematode management. Many available chemically-based nematicides do not provide long-term solutions for nematode suppression, and use is often restricted due to environmental and human health concerns. However, there is some evidence to suggest that plant-parasitic nematode management can be aided by cultural practices, biological control agents, and screening for naturally occurring nematicidal compounds in plants (Perez et al., 2003). Biologically-based nematicides can be an important tool for organic or farmers seeking to become more environmentally friendly, due to their ability to reduce chemical pressure in crop fields. Additionally, few existing nematicides are both biologically-based and consistently effective, leaving organic growers with limited options for management (Desaeger and Watson, 2019). Utilizing natural pest control strategies can both improve soil quality and human health by reducing harmful residue or contamination (Hulot and Hiller, 2021). To keep up with the demand of tomato production in the United States and the rest of the world, nematode populations can be monitored and managed through the use of a variety of integrated management strategies, when possible.

In recent years, an influx of biologically based nematicides have been developed and become commercially available to growers (Desaeger et al., 2020). Currently, there are a number of biologically based nematicides registered for use on tomatoes. However, product effectiveness varies greatly between growing season, moisture, soil composition, cultivar in field efficacy trials (Desaeger and Watson, 2019). The products involved in our experiments described below are developed by Microbes Inc. (MN21 and MN11) and are uniquely composed of lysed cell material (consisting of bacteria, yeast, and amino acids), with the goal of reducing plant-parasitic nematode populations.

To evaluate the efficacy of two new organic products (MN11 and MN21; Microbes, Inc.) at reducing *M. hapla* infestations, two greenhouse trials on greenhouse tomatoes (cv. Rutgers) were conducted. Furthermore, the first trial was repeated with slight modifications to confirm the efficacy of these potential products.

## Materials and Methods

### Colony Sourcing

*M. hapla* colonies were maintained in the MSU Plant Science Greenhouses on Michigan State University’s main campus. Root-knot eggs were collected from roots from an infested parsnip field in September 2019, and colonies were reared in gallon-sized polystyrene pots on tomatoes (cv. Rutgers). Inoculum (*M. hapla* eggs) for these trials were collected from tomato colonies using the NaClO root extraction process described in Hussey and Barker (1973).

### Greenhouse Trial Establishment – First Round

A greenhouse trial was established in 2021, to evaluate the potential efficacies of four product solutions against the plant-parasitic nematode, *M. hapla*. Steam sterilized soil was mixed with play sand at a 30:70 ratio, and approximately 500 cm^3^ of the soil mixture was placed into each of the 46 fresh polystyrene pots. Four seeds of a susceptible variety of tomato (cv. Rutgers) were seeded into pots. Four days later, the plants were thinned to two seedlings per pot. Any pots containing plants that contained less than two seedlings, or which showed signs of unthrifty growth, were removed prior to inoculation. Remaining pots were each inoculated with approximately 2000 *M. hapla* eggs each (N=36). One week following plant emergence, treatments were applied as a soil drench. Fifty milliliters of distilled water was applied to the noninoculated control pots. Velum Prime was applied to respective pots at a reduced rate equivalent to 6.5 oz/A. Next, 50 ml of respective solutions (MN11.1, MN11.2, MN21.1, MN21.2) were applied to the respective remaining pots, each with six replicates. Pots were labelled correspondingly and arranged in a randomized complete block design (RCBD) on the greenhouse bench and monitored weekly. Each week, plants were evenly fertilized with a 20-20-20 fertilizer solution to promote normal plant growth. Additionally, two weeks following product applications, plants were visually evaluated for signs of phytotoxicity. Following trial conclusion, above ground plant heights and weights were measured, and roots were separated from soil. Soil and roots were collected into plastic bags for further processing.

### Greenhouse Trial Establishment - Second Round

The experiment was repeated as previously described with slight modifications. The optimal formulations of each product were selected to repeat (MN11.2 and MN21.2). Inoculated, nontreated controls (positive controls) and noninoculated controls (negative controls) treatments were included for comparison. Each of the five treatments (positive control, MN11.2, MN21.2, Velum Prime, and the negative control) had twelve replicates. Six of the replicates were deconstructed at five weeks post-application (5 WPA), and the other six were concluded at ten weeks post-application (10 WPA). At both takedowns, the plant parameters (above ground height and root mass) and nematode parameters (juvenile nematodes/100 cm^3^ soil, mature female galls/1 g roots) described above were collected from each pot.

### Soil and Root Nematode Analysis

Soil within each bag was homogenized and a 100 cm^3^ volume subsample was collected for processing. Immediately following the trial conclusion, we used the sugar floatation centrifugal method to elucidate vermiform nematodes (Jenkins, 1964). Root-knot nematode juveniles were enumerated using a counting dish and an inverted Nikon TMS microscope at x200 and x1000 magnification, within five days of processing.

**Figure 1:**
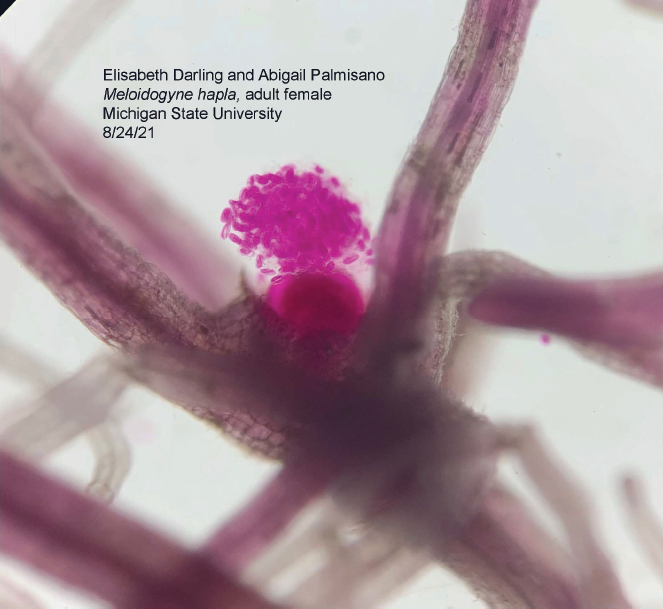
*Meloidogyne hapla* mature female with visible egg mass, stained using the NaOCl acid fuchsin-glycerin protocol. Roots collected 10 weeks post-inoculation from 1 g of positive control plants.

Roots were gently cleared of soil, rated on the Gall Indices Rating developed by Hussey and Janssen (2002): 0 indicating no galls and up to 5 indicating over 75% of the root system contains galls. Root systems were then weighed and recorded. Following evaluation, 1 g of lateral roots was collected from each plant. Each root sample was individually cut into 1 cm pieces and stained using the NaOCl acid fuchsin-glycerin procedure (Bybd et al., 1983). Samples were then temporarily stored in plastic petri dishes with glycerol until reading. From there, we used a dissecting scope at 35X to quantify galls within three days of staining (Fig. 1).

### Data analysis

Data analysis was completed via RStudio version 1.3.1093 (R Core Team, 2020). For each variable, a one-way ANOVA followed by Tukey’s HSD post-hoc analysis was conducted to determine significance between treatments for each plant and nematode parameter collected. Unless otherwise stated, *p*-values were obtained via a Tukey post-hoc analysis (a=0.05).

## Results

### Impact on plant parameters: phytotoxicity, root mass, and plant height

**Figure 2:**
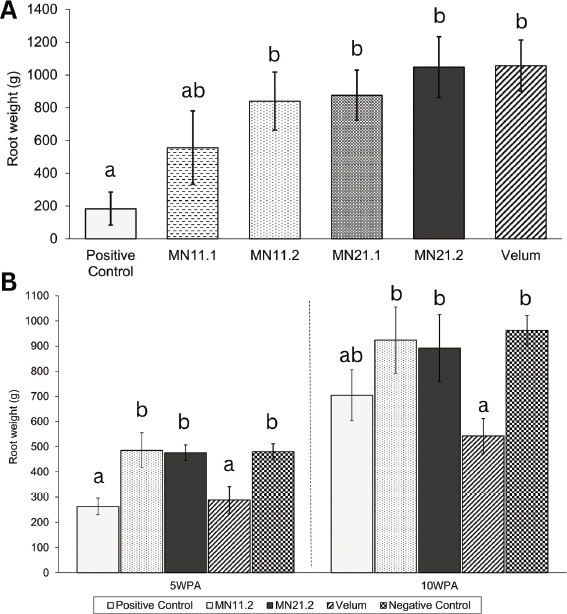
Average root mass of plants treated the first trial (A) and the second trial (B). Standard error bars reflect the standard error of the mean from each treatment. Unique letters indicate one treatment is significantly different from another (a=0.05).

During both trials, we did not observe any notable phytotoxic defects to plant coloration or growth for any of the treated plants. In the first trial, the positive control (inoculated, nontreated) possessed the lowest root mass of the five treatments. All of the positive control plants were heavily galled with lower masses (Figs. 2A,B). Root masses of plants treated with Velum Prime and MN21.2 were 5.76x and 5.72x heavier than the positive (inoculated, nontreated) control plants, respectively (*P* < 0.05). In the second experiment at 5 WPA, the positive control again possessed the lowest root mass. However, at 10 WPA, the root mass of the positive control was not significantly lower than any of the four treatments (Fig. 2B). The pots treated with MN21.2 were the only treatments to show significant increases on root mass in both trials. There were no significant trends observed from plant height from either trial (*P* > 0.05).

### Root Gall Index Rating for Nematode Infestation

**Figure 3:**
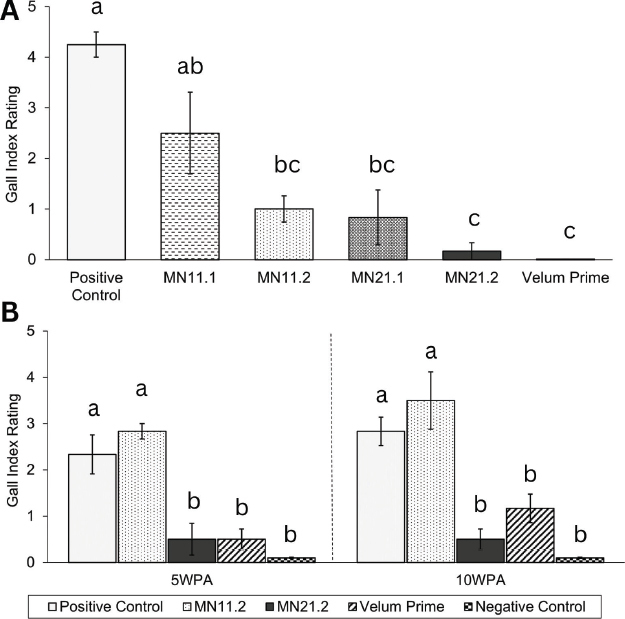
A,B. Gall Index Rating (GIR) of plants at trial takedown, ranked from 0 deeming no galling to 5 deeming severe galling of root systems. Standard error bars reflect the standard error of the mean from each treatment. Unique letters indicate one treatment is significantly different from another (a=0.05).

In the first greenhouse trial, plants with a higher Gall Index Rating (GIR) indicated more severe infestations and presence of galling on roots (Fig. 3A). Untreated (positive control) plants were significantly more galled than treatments M11.2, MN21.1, MN21.2, and Velum-applied plants (*P* < 0.01). In the repeated trial, plants treated with MN21.2 had a lower average GIR than both the inoculated positive control and plants treated with MN11.2 (Fig. 3B; *P* < 0.001). Plants treated with Velum Prime or MN21.2 possessed a significantly lower GIR than the positive control plants during both the first and second trials.

### Root-knot Nematode Concentrations

In the first greenhouse trial, all four treatments (MN11.1, MN11.2, MN21.1, and MN21.2) were lower than concentrations in the positive control plant roots. Both versions of MN21 were lower than MN11.1 (*P* < 0.05). In the second trial, MN21.2 reduced final (10 WPA) juvenile soil populations by 89.13% and mature female galls by 78.55% (*P* < 0.01). Velum Prime decreased final (10 WPA) juvenile soil populations by 90.08% and mature female galls by 79.51% (*P* < 0.01). MN21.2 significantly reduced female galls per 1 g roots for both trials (*P* < 0.01).

## Discussion

In the first greenhouse experiment, treatments MN21.1 and MN21.2 were both effective at reducing symptoms and signs of root-knot nematode infestations, similar to the application of Velum Prime (active: fluopyram; Bayer CropScience). The product MN11.2 showed potential efficacy in the first trial, but in the repeated greenhouse trial, plants treated with this product had higher galling than the untreated positive control plants. Studies on root knot nematode infestations at high concentrations on tomatoes are associated with increased root mass due to galling (Olthof and Potter, 1977; Gugino et al, 2006). However, our heavily galled positive control plants possessed root masses lower than plants treated with effective products and a negative control. Untreated, inoculated positive control plants also possessed visibly necrotic root tips along with their weight reduction. This could indicate that root losses in our infected plants may have occurred due to root tip necrosis, more than tissue increases from gall formation. *M. hapla* produces a higher number of “terminal” galls, or galls that incorporate the root tip, than other *Meloidogyne* species, which could explain why reduction occurred (Kinloch and Allen, 1972).

In the second experiment, our noninoculated, negative controls were contaminated with low rates of root-knot nematodes. This could have been due to droplet splashing during routine watering, or small amounts of soil transport on gloves during daily care. It is notable to mention that for the last two weeks of the repeat trial, the plants both from the positive control and MN11.2 pots were notably wilted.

Overall, our results indicate promising control of the northern root-knot nematode by the new nematicide, MN21.2. Results during both trials showed a similar reduction of mature female galls in roots and juveniles in soil populations to the plants treated with Velum Prime. These findings also consistent with prior studies in tomato plants that fluopyram is an effective product for reducing *Meloidogyne* spp. populations in tomato (Dahlin et al., 2019; Desaeger and Watson, 2019). Despite these promising findings, greenhouse nematicide trials involve a uniform, well-controlled environment which limit many confounding variables apparent under field conditions, so further evaluation under field settings is necessary in determining true product efficacy. However, the consistency of our findings between the repeated trials suggests that MN21.2 is an effective tool for managing the northern root-knot nematode. If this product is found to have consistent results under field evaluation, it could provide tomato growers with an effective organic management option when facing northern root-knot nematode infestations.
